# A 4.8-μV_rms_-Noise CMOS-Microelectrode Array With Density-Scalable Active Readout Pixels via Disaggregated Differential Amplifier Implementation

**DOI:** 10.3389/fnins.2019.00234

**Published:** 2019-03-21

**Authors:** Jun Ogi, Yuri Kato, Yusaku Nakashima, Kenji Ikeda, Motoko Jingu, Yoshihisa Matoba, Naohiko Kimizuka, Chigusa Yamane, Masataka Maehara, Takuya Kishimoto, Shigeki Hashimoto, Eriko Matsui, Yusuke Oike

**Affiliations:** ^1^Research Division 1, Sony Semiconductor Solutions Corporation, Kanagawa, Japan; ^2^Department of Biomedical Research, R&D Center, Sony Corporation, Tokyo, Japan; ^3^Research Division 2, Sony Semiconductor Solutions Corporation, Kanagawa, Japan

**Keywords:** microelectrode array (MEA), CMOS integration circuits, readout noise, differential amplifier circuit, neuron action potentials

## Abstract

We demonstrate a 4.8-μV_rms_ noise microelectrode array (MEA) based on the complementary-metal-oxide-semiconductor active-pixel-sensors readout technique with disaggregated differential amplifier implementation. The circuit elements of the differential amplifier are divided into a readout pixel, a reference pixel, and a column circuit. This disaggregation contributes to the small area of the readout pixel, which is less than 81 μm^2^. We observed neuron signals around 100 μV with 432 electrodes in a fabricated prototype chip. The implementation has technological feasibility of up to 12-μm-pitch electrode density and 6,912 readout channels for high-spatial resolution mapping of neuron network activity.

## Introduction

In previous decades, a complementary-metal-oxide-semiconductor (CMOS)-based MicroElectrode Array (MEA) was introduced to achieve two-dimensional high spatial resolution mapping of action potentials(APs) (Eversmann et al., 2003; Ballini et al., 2014). The high resolution mapping of APs in neuron cells can provide information regarding the complexities of neuron network activities, such as the nature of dendritic integration, the electrical functions of dendritic spines, and variations in spontaneous native activity patterns or network oscillations (Gross et al., 1997; Peterka et al., 2011).

To better comprehend the network activity, both readout channel numbers and electrode density must be further increased while maintaining sufficiently low noise levels (less than 10 μV_rms_). Various implementation methods have been proposed in previous works on the CMOS-MEA (Obien et al., 2015). While readout techniques with Active Pixel Sensors (APS) have been proposed to increase the channel number to over ten thousand, electrode density is limited above electrode pitch of 30 μm, because of the large area of the readout circuits integrated under the each electrode (Huys et al., 2012; Johnson et al., 2013). We present scalability for a higher-density and larger channel number with a 24-um-pitch and 6,912-readout-channels CMOS-MEA (Ogi et al., 2017). However, the noise level was 23 μV_rms_ in this CMOS-MEA, and this was not sufficiently low to observe the neuron APs.

In this paper, we demonstrate a 4.8-μV_rms_ noise CMOS-MEA based on the APS readout technique with disaggregated differential amplifier implementation method. This has the technological feasibility for high-density electrode integration with 12-μm pitch electrode density and 6,912 readout channels.

## Implementation

**FIGURE 1:**
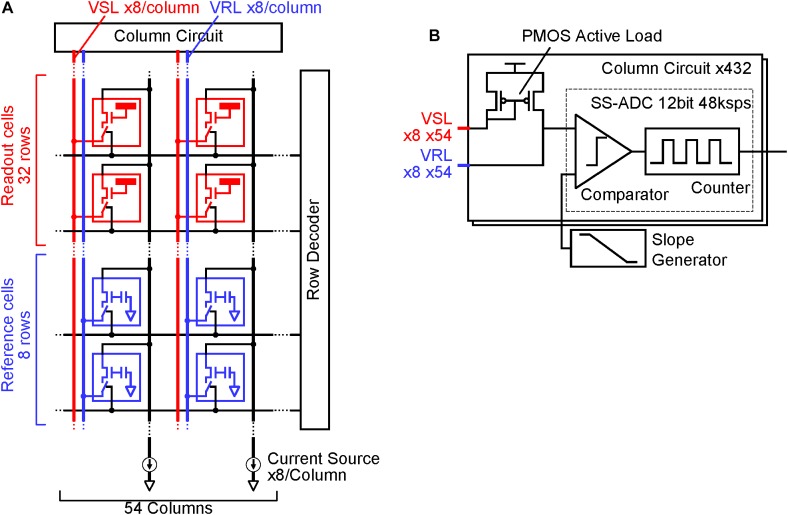
CMOS-MEA implementation: **(A)** Readout pixel array, reference pixel array and column circuit connection. The reference pixel array (Blue) is implemented in addition to the readout pixel array (Red), column circuit and current source. The readout pixels and reference pixel are connected to the column circuit via vertical signal line (VSL) and vertical reference line (VRL) by the selection signal from the row decoder. **(B)** Details of each column circuit. Each column circuit includes a pair of PMOS active loads and a single slope ADC. The combination of one readout pixel, one reference pixel and a pair of PMOS active load acts as a differential amplifier, which amplifies the input signal at the readout pixel before input to ADC.

To reduce noise in the readout channel of CMOS-MEAs, the channel is usually equipped with a large input capacitance, a low-noise and high-gain differential amplifier, and a band pass filter (Frey et al., 2010). In previous works for sub-10 μV_rms_ readout noise using the APS, these circuit elements were integrated under each electrode, increasing the readout pixel area (Johnson et al., 2013). Figure 1 shows the readout implementation of this work. The circuit elements in the differential amplifier are divided into the readout pixel, the reference pixel and the column circuit in this implementation; we call this disaggregated differential amplifier.

In addition to the readout pixel array and the column circuits, the reference pixel array is implemented. Electrodes that can sense cell AP are directly connected to the input amplifier in the readout pixel. In the reference pixel, the input amplifier connects to the ground via an input capacitance. When each one row in the readout and reference pixel array is selected by a signal from the row decoder, the two input amplifiers are connected to the PMOS active load in the column circuit via vertical signal line (VSL) and vertical reference line (VRL), and the differential amplifier is constituted by the connection. This differential amplifier amplifies the AP signals from the sensing electrode, and it contributes in relatively reducing the input-referred-noise induced by analog to digital converters (ADCs) after the amplifier. Despite the simplicity and small area of the readout pixel circuit, the high-gain differential amplifier can be implemented by the disaggregation of the circuit components in each block.

**FIGURE 2:**
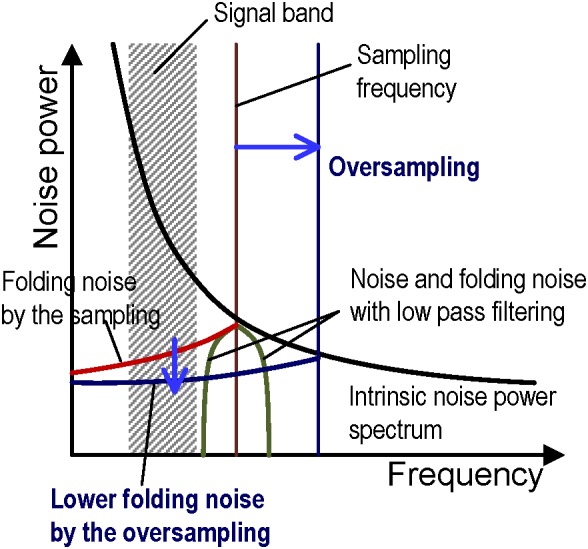
Conceptual diagram of folding noise reduction by oversampling with the noise power spectrum vs. frequency. The noise is folded at the sampling frequency (red vertical line) after analog to digital conversion and it increases the total noise in a signal band (red curve). A low pass filter is generally utilized to remove the folding noise in the signal band (green curve). In this work, oversampling shifts the folding point at a higher frequency (dark blue vertical line) and it can reduce the folding noise in the signal band because the noise power gradually decreases as frequency increases.

Single slope ADCs (SS-ADCs) have the advantage of high-speed data conversion through the use of a high-speed input clock (Wakabayashi et al., 2010) and it performs 48-ksps (sample per second) high-speed ADC with 12-bit resolution in this work. The number of VSLs and column circuits are eight times larger than the pixel column numbers, as shown in Figure 1, so that the 8-rows and 54-columns pixel array (total 432 pixels) can be read simultaneously with the 432 parallel VSLs and column circuits at 48-ksps. In contrast, the required signal frequency band to measure neuron AP (AP band) ranges from 300 Hz to 3.3 kHz (Yuan et al., 2016), and the required minimum sampling rate is about 6 kHz. The 48-ksps sampling rate, which is eight times larger than the 6 kHz, contributes in reducing the folding noise in the ADC, as shown in Figure 2. Higher frequency noise is folded at the half of the sampling rate, and the folding noise increases the noise power in the signal frequency band. A low pass filter is usually utilized to cut the folding noise. On the other hand, the noise power is gradually decreased as frequency is increased and over sampling can shift the folding point of the noise to lower noise power. As a result, oversampling can reduce the folding noise instead of the low pass filter. This also contributes in decreasing the pixel area, due to the absence of the low pass filter in each pixel.

## Prototyping

**FIGURE 3:**
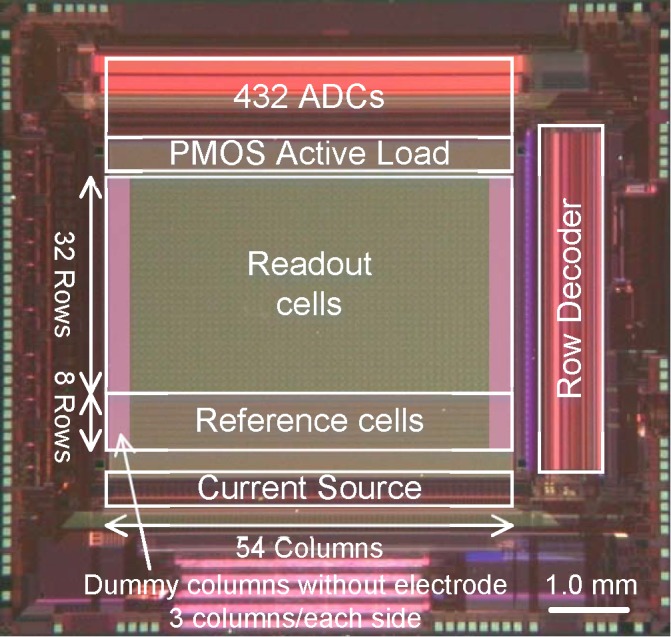
Micrograph of the fabricated prototype chip. The prototype chip has 32 row and 54 column readout pixel array, 8 row and 54 column reference pixel array and 432 column circuit. The column circuit pitch is 12 μm and the readout electrode pitch is 96 μm. Platinum electrodes are integrated on the area of the readout pixels with 24 μm pitch, by extending the CMOS backend process following formation of an aluminum top metal layer.

Figure 3 shows a micrograph of a fabricated prototype chip with a 0.14-μm 1-poly 3-Cu 1-Al CMOS process. The column circuit pitch is 12 μm and the readout electrode pitch is 96 μm. Although the electrode pitch is limited by the number of the column circuits, the readout circuit area in each pixel is less than 81 μm^2^. This is considerably smaller than the 450 μm^2^ in the previous two-stage amplifier implementation (Huys et al., 2012). The 432 column circuits were integrated in the small area over the readout pixel array because of the area efficiency and the high-density integration of single slope ADCs. The small readout circuit area and small column pitch contribute to the high-scalability of the CMOS-MEA in this work.

Platinum electrodes are integrated on the area of the readout pixels with 24 μm pitch, by extending the CMOS backend process following formation of an aluminum top metal layer. Selected electrodes with 96 μm pitch are connected to the active readout pixels one by one and work as the sensing electrodes.

## Measurement Results

### Random Noise on Readout Circuit

**FIGURE 4:**
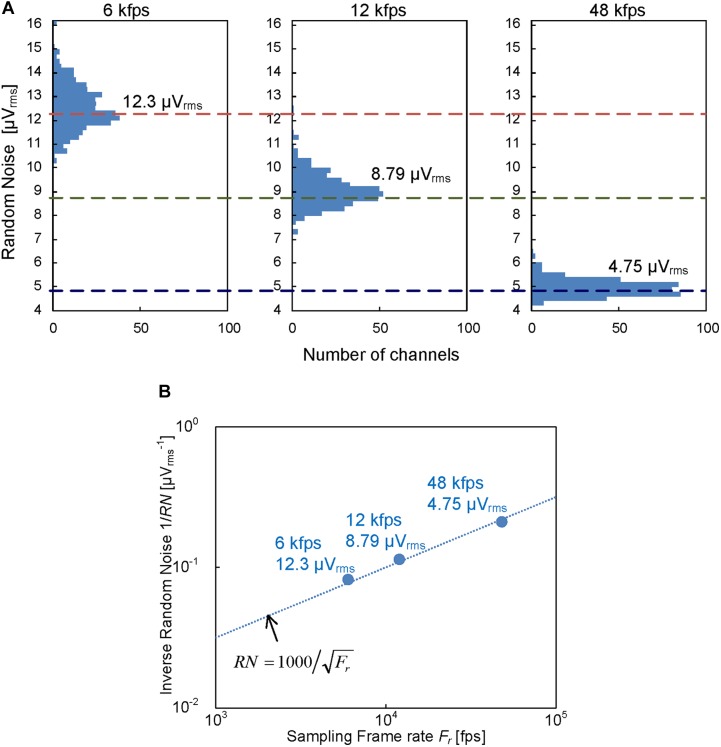
**(A)** Readout random noise distribution with different sampling frame rate; 6 kfps, 12 kfps, and 48 kfps. Each noise distribution was measured with 384 readout channels in the prototype chip and the corresponding median values are 12.3, 8.79 and 4.75 μV_rms_, respectively. **(B)** Random noise dependence on sampling frame rate. The frame rate dependence is proportional to the inverse of the frame rate.

Figure 4 shows that the input-referred noise on the readout channels in the prototype CMOS-MEA was reduced to 4.75 μV_rms_ after post-processing of the frequency band limitation to the AP band by digital finite response (FIR) filters. The intrinsic noise without FIR filtering was reduced to 12.8 μV_rms_ by the disaggregated differential amplifier implementation. In addition, the noise after filtering was further reduced by the over sampling in the ADCs, as shown in Figure 4A. The reduction is proportional to the inverse of the readout sampling rate, as shown in Figure 4B. These results show that the disaggregated differential amplifier and the oversampling can reduce the random noise as we intend.

### Neuron Action Potential

**FIGURE 5:**
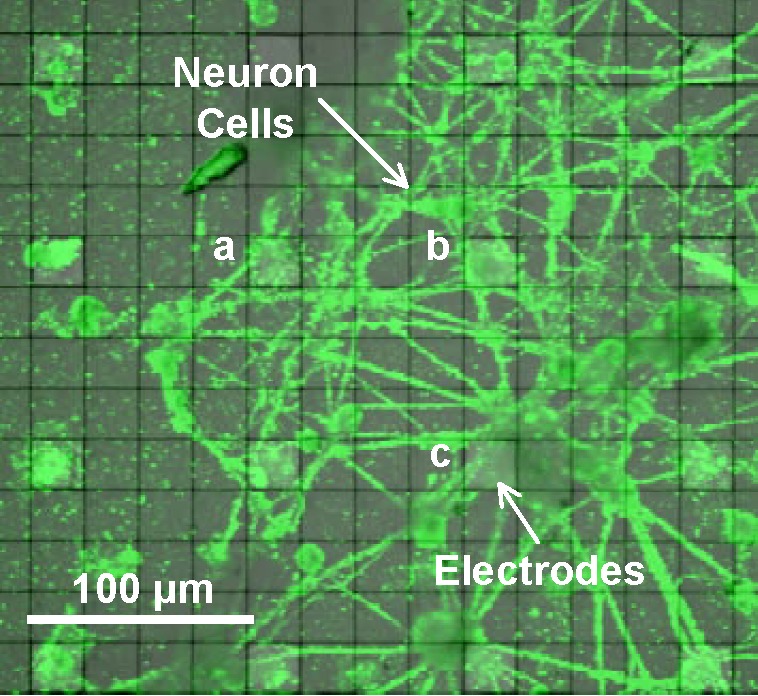
Optical micrograph image of neuron cells cultured on the prototype CMOS-MEA. A fluorescence image of the neuron cells is composited on the visible image of the microelectrode array. The green shapes are cultured neuron cells and the gray bright regions under the neurons are the sensing electrodes.

Figure 5 is a visible optical micrograph of the prototype CMOS-MEA with composited fluorescence imaging of neonatal pyramidal neurons primary cultured on the prototype chip. The gray bright regions under the green neurons are the sensing electrodes. The neurons were cultured on the electrode coated with PDL and Laminin for 4 weeks in 37°C 5% CO_2_. PKH67 Green Fluorescent Cell Linker Midi Kit for General Cell Membrane Labeling was used to capture the fluorescence imaging of the neuron cells.

**FIGURE 6:**
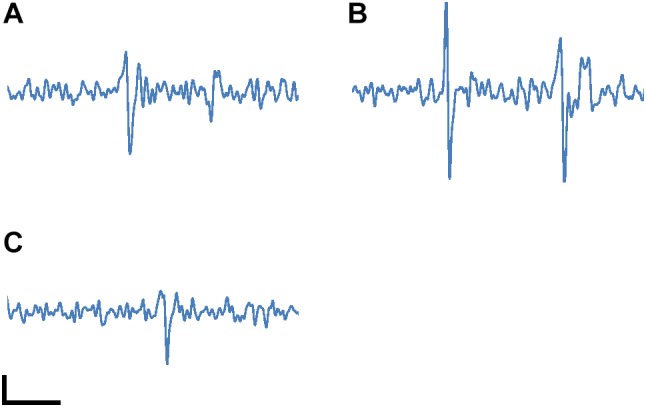
Measured neuron action potential signals with the electrodes **(A)**, **(B)** and **(C)** marked in Figure 5. The spikes in the signals correspond to spontaneous APs from the cultured neurons. The spikes were observed with Glutamate stimulating during the measurement. The horizontal and vertical scale bars are 5 ms and 50 μV, respectively.

Figure 6 shows action potential signals observed with electrodes (a), (b) and (c) covered by the cultured neurons in Figure 5. The spikes in the signals correspond to spontaneous APs from the cultured neurons with Glutamate stimulating. 100 μV peak signal levels were clearly recognized because of the sufficiently low noise in the readout circuit. These results show the feasibility of our CMOS-MEA technology for neuron AP measurements.

## Scalability and Comparison With Previous Work

The electrode density and channel number can both be increased by increasing the ADC number and pixel multiplication factor for each ADC, due to the small readout pixel area and small column pitch in the CMOS-MEA technology proposed in this work.

**FIGURE 7:**
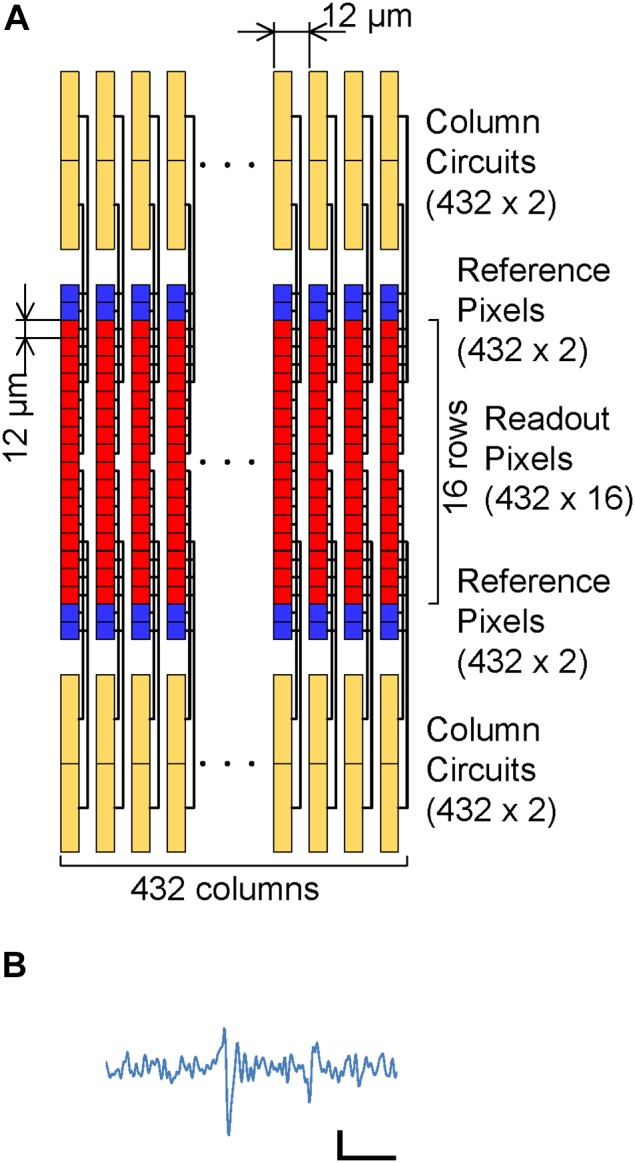
**(A)** Possible scaling with four-tier column circuits (1,728 column circuits) on both sides of the pixel array and four times multiplication (12 kfps frame rate). The electrode pitch will be decreased to the column pitch (12 μm), with 6,912 readout channels (16 rows and 432 columns) by the increasing of the column circuits number and the sequential readout with four times multiplication. **(B)** Emulated action potential signal with 12 kfps sampling rate. This signal was generated by decimating the sampling point from the original signal of Figure 6A before FIR digital filtering. The horizontal and vertical scale bars are 5 ms and 50 μV, respectively.

Figure 7A shows the case of four-tier column circuits (a total of 1,728 column circuits) on both sides of the pixel array with four times multiplication. The larger number of column circuits, compared with the prototype in the present work, contributes to an increase in the readout electrode number and a reduction in the electrode pitches, even with the same in-pixel and column circuit implementations as those in the present work. In the present work, the column pitch limits the readout electrode pitch, because the in-line eight column circuits (96 μm pitch) are needed to simultaneously read eight rows. On the other hand, for the four-tier case, four rows of the pixel array with a 12 μm pitch can be read simultaneously by the four-tier column circuits and four parallel VSLs. In addition, the other four rows in the pixel array are connected to the same VSL and column circuit, and the connected rows are sequentially read with the selecting signals from the row decoder (four times multiplication). This quadruples the readout row number to 16, while it quarters the sampling frame rate to 12 kfps. As a result of the four-tier column circuits and four times multiplication, the readout electrode pitch is decreased to the column pitch (12 μm) and 6,912 electrodes (16 rows and 432 columns) can be read at 12 kfps.

Although a small pitch of the electrode reduces the available area for the in-pixel readout circuits and affects the readout noise level, the noise level is still maintained because the area of each in-pixel circuit in this work was smaller than the area for 12 μm square ( = 144 μm^2^), as mentioned in “Prototyping.” On the other hand, the low sampling frame rate (12 kfps) should have increased the AP band noise to 8.8 μV_rms_, as shown in Figure 4B, but the 100 μV signal peak is still observed, as shown in Figure 7B, because the noise level was still less than 10 μV_rms_. In addition, increasing the column circuit numbers should result in an increased chip size, however, the chip size is limited due to the small area of the ADCs, as can be seen in Figure 3.

Furthermore, the introduction of advanced processes for logic circuit in the column circuit can increase the channel number and decrease readout random noise. In single slope ADCs, the area and the sampling speed are limited by the transistor size for digital counter. However, the area can be decreased and the sampling speed can be increased by reducing the transistor size in the advanced CMOS processes. The small area contributes in increasing ADC numbers and readout channel numbers, and the higher sampling speed contributes in decreasing the readout random noise.

**FIGURE 8:**
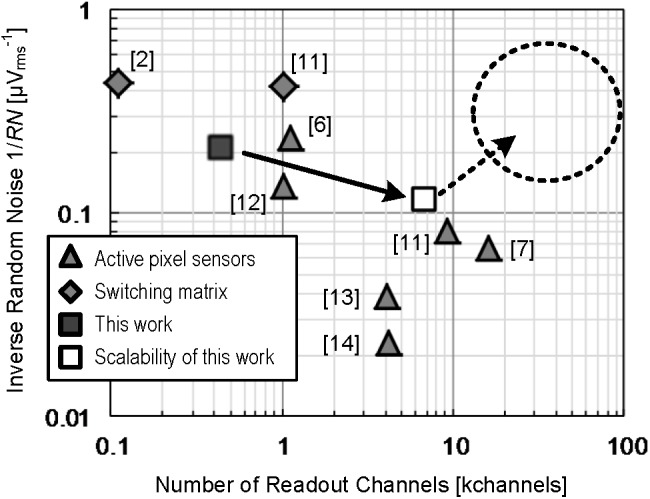
Comparison of this work with previous works for high-density CMOS-MEA in terms of the relations between readout channels number and readout noise. The shaded triangles present the previous works on APS readout scheme and the shaded diamonds denote the previous works on switching matrix readout scheme. The closed square presents the result of this work. The opened square indicates possible scaling applying the CMOS-MEA technology proposed in this work. The area in the dashed circle is also feasible to achieve by applying more advanced CMOS processes with our CMOS-MEA readout scheme.

Figure 8 shows the comparison of this work with previous works for high-density CMOS-MEA by relationships between readout channels number and readout noise. The noise in this work is one of the lowest levels in the previous works of the APS readout scheme (Imfeld et al., 2008; Huys et al., 2012; Johnson et al., 2013; Bertotti et al., 2014; Yuan et al., 2016; Lopez et al., 2018) and it is comparable with the previous works of switching matrix readout scheme (Ballini et al., 2014; Yuan et al., 2016), which has an advantage usable in readout noise reduction. The largest number of the channels in previous works is also feasible with the four-tier ADC implementation by applying the technology proposed in this work. Further scaling is also feasible up to 100 thousands readout channels with utilizing more advanced CMOS processes.

## Conclusion

We introduce the disaggregated differential amplifier implementation that can reduce the circuit area of the readout pixel in CMOS-MEA with APS readout technique. The prototype chip with the implementation demonstrated a 4.8-μV_rms_ readout noise and observation of neuron AP at about a 100 μV signal level. The CMOS-MEA technology proposed in this work is scalable for high spatial resolution mapping of neuron network activity up to 100 thousands readout channels.

## Data Availability

The datasets generated for this study are available on request to the corresponding author.

## Author Contributions

JO, YK, MJ, YM, NK, CY, MM, and YO contributed to the CMOS circuit design and prototyping, and circuit characteristics evaluation. YN, KI, TK, SH, and EM contribute to observation of the neuron action potential with the prototype chip.

## Conflict of Interest Statement

All authors were employed by Sony Corporation. JO, YK, MJ, YM, NK, CY, MM, and YO were also loaned employee in Sony Semiconductor Solutions Corporation.
